# Pipe Dreams: Tapping into the Health Information in Our Sewers

**DOI:** 10.1289/ehp.124-A86

**Published:** 2016-05-01

**Authors:** Carrie Arnold

**Affiliations:** Carrie Arnold is a freelance science writer living in Virginia. Her work has appeared in *Scientific American*, *Discover*, *New Scientist*, *Smithsonian*, and more.

Most of us have an attitude of “flush it and forget it,” but to scientists like Rolf Halden, our waste is a bonanza of valuable information on population-level chemical exposures. Halden is an environmental scientist at Arizona State University’s Biodesign Institute, where he maintains the National Sewage Sludge Repository—a collection of hundreds of samples of raw sewage and sludge collected from more than 200 sites around the United States.^1^

The sample bottles are filled with a brownish-black slurry derived from raw sewage, “so it has everything that is flushed down the toilet,” says Arjunkrishna Venkatesan, a postdoctoral research associate on Halden’s team. “If you collect samples from sewerage manholes, sampling equipment is going to get clogged, and you’re going to have hair, condoms, and all sorts of stuff.” The laboratory staff filter out most of those solids, then freeze the samples for storage.

**Figure:**
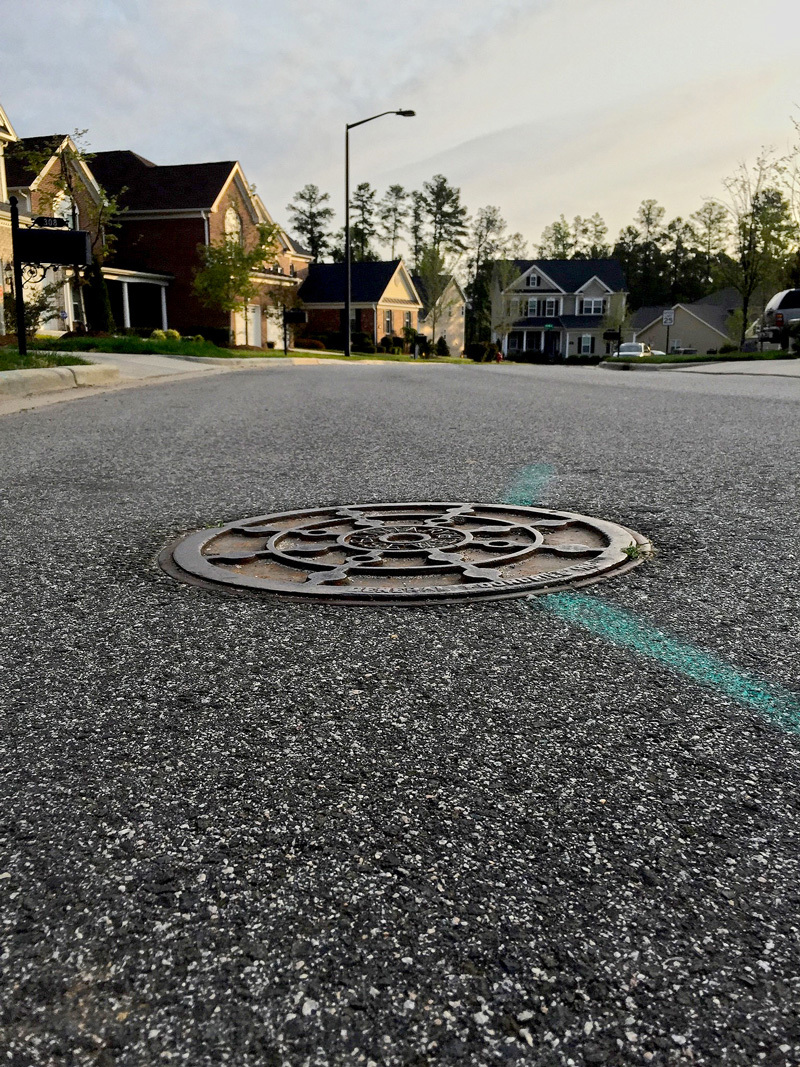
The emerging field of sewage chemical-information mining is taking advantage of a readily available yet underappreciated resource: the untreated waste flowing under our feet and the biosolids remaining after treatment. It turns out that sewage and sewage sludge hold a wealth of data on chemical consumption and exposure, and potentially even the health status of whole communities. EHP

As disgusting as the samples may be, the repository is helping to open up a field known broadly as sewage chemical-information mining (SCIM). This field is using the sewage destined for wastewater treatment plants as a medium for chemical exposures within a community, exposures that are impractical to measure by other means. Other studies are looking at sewage sludge (the solids that remain after wastewater treatment) for information on chemicals that can accumulate in the human body. Still another approach, known as BioSCIM, measures biomarkers in sewage as a way to assess the overall health status of communities.

SCIM initially took off in 2001 when scientists hypothesized they could measure the metabolites of illicit drugs like cocaine, heroin, and methamphetamine in untreated wastewater collected from city sewers.^2^ This particular application of SCIM was originally known as “sewage epidemiology.” The success of this technique has been tested in Europe^3^^,^^4^ and most recently provided per capita estimates of drug usage in cities including Antwerp, Stockholm, and London.^5^

Now sewage epidemiology is being applied to substances beyond illicit drugs. Since 2010 Halden has published nearly 50 papers on the subject. One study calculated per capita chemical consumption and, based on levels found in sewage sludge, estimated exposure to more than 70 pharmaceuticals and other chemicals used in consumer products.^6^ “This work lets us put a finger on the chemical pulse of a nation,” Halden says.

## Surveying Sludge

As part of the passage of the Clean Water Act in 1972, the U.S. Environmental Protection Agency (EPA) established requirements for safely disposing of sewage sludge and assessing the nation’s sludge to stay on top of the chemicals it contains.^7^ That’s especially important for treated sewage sludge, which is a popular fertilizer for agricultural fields. To date, the EPA has conducted four national surveys, most recently testing samples of sewage sludge from municipal wastewater treatment plants for dioxins, pharmaceuticals and personal care products (PPCPs), brominated flame retardants, nine different heavy metals, bacteria, viruses, and parasites.^7^

**Figure:**
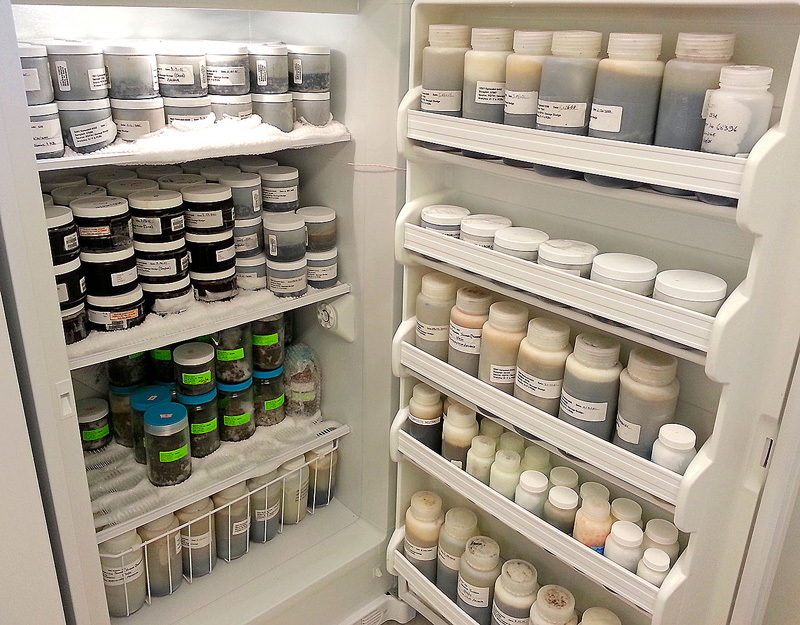
Freezers at the National Sewage Sludge Repository hold hundreds of samples of raw sewage and sludge collected from more than 200 sites around the United States. © Arjunkrishna Venkatesan

Awareness of PPCPs as a potential concern began with a 1999 report by Christian Daughton, a senior research scientist at the EPA, and colleague Thomas A. Ternes. They pointed out that sewage was a conduit by which low levels of these chemicals were entering the environment virtually unnoticed. Moreover, it was becoming clear that PPCPs and other so-called contaminants of emerging concern—those chemicals that are being detected for the first time or in increasing amounts—tend to elude treatment and remain in treated water and sewage sludge, returning to the environment and in some cases accumulating in living organisms, including humans.^8^

“There were chemicals that we didn’t know were there, that we didn’t know could be a health concern, and that we didn’t think to analyze,” Daughton says. “Sewage was mostly ignored—it’s not seen as being actually in the environment, and it’s not pleasant to work with.”

Measuring human exposure to all commercially available chemicals is impossible. Testing for even a handful can be prohibitively expensive, and fully understanding the body burden of these chemicals would require blood, urine, hair, and fecal samples from thousands of individuals. Wastewater and residual sludge, however, contain urine and feces from large numbers of people, and there is plenty of it. And since the samples can’t be tied to individuals, researchers don’t have to worry about obtaining informed consent from subjects or approval from institutional review boards.

The EPA began testing for contaminants of emerging concern in the 2001 National Sewage Sludge Survey, gathering samples from 94 wastewater treatment plants from 32 states and the District of Columbia.^9^ But eventually the EPA had more samples than it could use. When Halden, then at the Johns Hopkins School of Public Health, was asked if he might be interested in taking charge of the leftovers, he jumped at the chance. “I realized that what they had, although smelly, represented a human health observatory,” Halden says.

## Putting Sludge to Work

Since 2001, Halden had been studying the widely used antimicrobials triclosan and triclocarban. In 2002, millions of pounds of triclosan and triclocarban were added to everything from toothpaste to plastics.^10^ Halden’s work in the Baltimore metro area had indicated that triclocarban was, at that time, probably one of the top 10 most frequently occurring organic water contaminants.^11^ The market for these products has been estimated at $886 million,^12^ but publicly available production data for these and other commercially important chemicals are too vague to use in determining year-to-year trends in chemical consumption, Halden says. (Meanwhile, his work is contributing to these compounds being phased out of use in many products.)

What’s more, no one knew exactly what was happening to these products in the environment, how much might be remaining in treated wastewater, how much was in the biosolids being used as fertilizer on agricultural land, and how much humans were actually exposed to in their daily lives. Halden believed samples from the National Sewage Sludge Repository could provide some of those answers.

The wastewater treatment process involves a variety of physical, chemical, and microbial processes to remove objects from wastewater (everything from toilet paper and tampons to dead animals) and eliminate organic matter, harmful microbes, and other contaminants. The water is then suitable for further treatment to become drinking water.^13^

**Figure:**
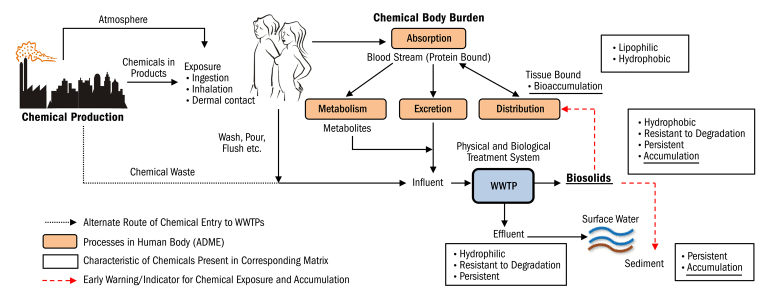
Most chemicals that people are exposed to via consumer products are ultimately washed off or excreted; they are collected in municipal sewers and sent to wastewater treatment plants (WWTPs). Fat-soluble chemicals—i.e., those that can accumulate in the human body—tend to be the ones that resist treatment. Courtesy Arizona State University

Given that this process is intended to degrade many chemicals, Halden wasn’t sure if he could find measurable amounts of these antimicrobials in the sewage sludge. But a preliminary analysis published in 2010 revealed that not only were triclosan and triclocarban present, their amounts were far higher than Halden expected, accounting for two-thirds of the PPCPs his team measured in biosolids. The two chemicals amounted to between 210 and 250 metric tons of product found in sewage sludge around the country each year, chemicals that were then reapplied to farmland in fertilizer^14^ and were found to persist in the soil over time.^15^

Halden knew from the start that simply measuring the amounts of triclosan and triclocarban in sludge wouldn’t tell him accurately how much humans had been exposed to. Many of the products that contained these two antimicrobials—such as soaps and toothpastes—were designed to be washed down the drain after contact with the human body. So he tested raw and treated sewage sludge for metabolites of triclosan and triclocarban, such as 2'-hydroxy-3,4,4'-trichlorocarbanilide, which would more accurately assess human exposure via ingestion, inhalation, and skin exposure.^16^

Halden and colleagues used liquid chromatography–tandem mass spectrometry to measure the amount of the antimicrobial metabolites in the sludge samples. Next they back-calculated the amount the average person was exposed to, based on the number of people served by the wastewater treatment plant. They adjusted for industrial, agricultural, and commercial contributions to wastewater as well as any recent weather events (in the eastern United States, storm sewers and sanitary sewers are generally combined before reaching the treatment plant, so rainfall dilutes the sewage; in the West, they’re generally kept separate^17^).

“Even when looking at the consumer compounds themselves, rather than their metabolites only, there was a remarkably strong positive linear relationship between both the types and amounts of chemicals we found in sewage sludge and what the CDC [Centers for Disease Control and Prevention] found in human testing, which says that this technique can provide information on chemical body burdens,” Venkatesan says. “This placed in our hands a real-time screening tool to narrow down what to test for, and to bring to light in an inexpensive fashion the chemical composition and potential exposures of millions of people.”

In addition to triclosan and triclocarban, Halden and Venkatesan discovered in the sludge some 121 chemicals listed as contaminants of emerging concern. About 70% of the chemicals detected in the sludge had also been measured in human tissue samples collected as part of the National Health and Nutrition Examination Survey (NHANES).^6^ The researchers had some of their first evidence that sewage sludge could provide reasonably accurate measurements of human exposures to environmental contaminants.

## Broadening the Search

The advantages of sewage epidemiology methods over traditional methods of assessing human exposure to environmental contaminants were becoming clear. As part of NHANES, the CDC collects urine and blood specimens to measure everything from metals like lead, copper, arsenic, and mercury to the popular insect repellant DEET. In the world of environmental studies, NHANES is big—since its inception it has surveyed over 140,000 Americans.^18^ But the data collected from the 2006–2007 Targeted National Sewage Sludge Survey provided information on chemical measurements that the authors said could be extrapolated to more than 3,300 wastewater treatment plants serving millions nationwide.^7^ With these advantages in mind, and his early successes looking at triclosan and triclocarban in sewage sludge, Halden and postdoc Venkatesan began to expand the list of chemicals they looked for.

When it comes to selecting which compounds to study in sewage and sludge samples, scientists can’t just blindly begin testing samples. For one thing, not all chemicals make their way into sludge, especially pharmaceuticals that are highly water soluble. Those chemicals that tend to be found in sludge are more likely to be hydrophobic, and therefore fat soluble. “Whatever chemicals are left in the sludge are the persistent ones. They do not degrade in the sewer lines or in the wastewater treatment plants, and so [they] can accumulate in sludge,” Venkatesan says. “And chemicals that can accumulate in sludge can also potentially accumulate in the human body.”

**Figure:**
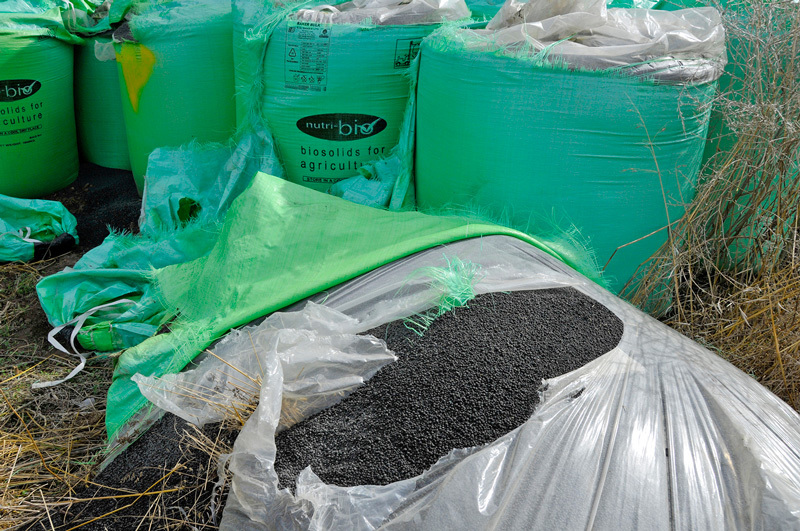
Persistent substances that withstand treatment can find their way into fertilizer derived from sewage sludge. This popular soil amendment for agricultural fields thus represents a potential route for anthropogenic chemicals to enter the food supply. © Justin Kase zsixz/Alamy

Some of the first compounds on Halden’s list were other PPCPs, a laundry list of prescription medications (including antibiotics, antidepressants, and statins), over-the-counter medications (acetaminophen, ibuprofen), and surfactants (including alkylphenols and their ethoxylates, and perfluorinated compounds). As the consumption of drugs increased with the number of prescriptions written, Halden found, so did the potential for these chemicals to build up in the water supply and in biosolids. Findings from the 2006–2007 National Sewage Sludge Survey showed that antibiotics including ciprofloxacin had been in the environment for a long time and were present at relatively stable levels across the country.^14^

As with triclosan and triclocarban, these initial sludge data told Halden how much of these chemicals might be returned inadvertently to soil.^19^^,^^20^ And for chemicals that persisted during treatment and accumulated in sludge, it also provided clues on human exposure and other risks, such as the potential for development of antibiotic resistance in microorganisms native to soils receiving sludge applications.^21^

Halden and his team continue to broaden the suite of chemicals they are looking for, and they have set up a system to share sewage and sludge samples with collaborators worldwide.^1^ For instance, Kurunthachalam Kannan, an environmental scientist at the New York State Department of Health, worked with Halden and other scientists to show that bisphenol A and its chemical replacements could be found in sludge, although the amounts that persisted in biosolids would reflect only a tiny fraction of a human’s average exposure.^22^

Halden’s group also detected *N*-nitrosamines,^23^ carcinogens that can arise as by-products of chlorination during wastewater treatment,^24^ from residential sources,^25^ and from the production of rubber consumer goods.^26^ In other studies, they measured levels of brominated flame retardants^27^ and their derivative contaminants, which include polybrominated dibenzo-*p*-dioxins and dibenzofurans.^28^ Halden and Venkatesan have documented the presence of perfluoroalkyl substances^29^ (persistent, toxic chemicals used in a variety of industrial, military, and firefighting processes^30^) and alkylphenol ethoxylates^31^ (widely used endocrine-active commercial and household detergents).^32^

It is especially worrisome that relatively high concentrations of these compounds were readily found in biosolids from across the United States, because it creates a positive feedback loop, says Jörg Drewes, a water systems engineer at the Technical University of Munich in Germany. High human exposures mean higher concentrations in biosolids, and with the use of biosolids as fertilizer, humans have an additional route of exposure.

“The simple paradigm in drinking water supply is to provide separation between sewage and [treated] drinking water. It seemed to work for decades, but then the analytical chemistry became more sensitive, and we could look at a different level and see that some of these chemicals were showing up in the drinking water supply,” Drewes says.

## Opening Up New Possibilities

According to Halden and Vankatesan, scientists have only begun to scratch the surface of what can be learned from this type of research. The chemical exposures measured in NHANES and similar surveys require that scientists know what they are looking for. The high sensitivity and specificity of liquid chromatography–tandem mass spectrometry means that researchers can conduct an unbiased search for chemicals in sewage sludge that may be present in unusually high amounts. When Halden and Vankatesan did just that, they were able to track 26 chemicals previously unmonitored in U.S. biosolids,^33^ as well as identify a variety of potentially problematic chemicals that are produced in high volumes.^34^

Other scientists are taking a new direction. In an unpublished study, a team led by Italian researcher Sara Castiglioni of the Mario Negri Institute for Pharmacological Research in Milan has been analyzing the relationship between days with high levels of air pollution and amounts of albuterol and metabolites found in untreated wastewater. Albuterol is a medication used in asthma inhalers. Castiglioni’s work thus uses measurements of these substances at different points in time as a proxy for measuring increases in asthma symptoms. “We are trying to see whether the use of bronchodilators corresponds to days with high particulate matter in the air,” she says.

The work is not without its limits, however. Instead of estimating an individual’s exposure levels, it captures a per capita level that is a community average. Information gleaned about a community’s chemical exposures also has an inherent amount of uncertainty in it, as the population is in a constant state of flux. People move away, others come to the area for business, and heavy rains or melting snow can affect the amount of water in the sewage system.

**Figure:**
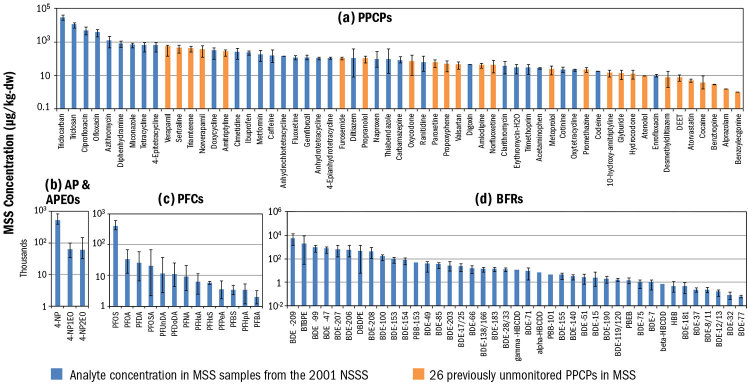
Surveys of sewage sludge have revealed varying concentrations of dozens of anthropogenic chemicals, including a) compounds used in pharmaceuticals and personal care products, b) surfactants, c) perfluorinated compounds, and d) brominated flame retardants. Analysis of samples archived at the National Sewage Sludge Repository has revealed the presence of previously unmonitored compounds (orange bars). Source: Venkatesan et al. (2015)^1^

“We need to distinguish between levels of occurrence and levels of exposure,” Drewes says, because unmetabolized “parent” chemicals measured in the sewage system can also reflect situations such as people flushing unused medications down the toilet.

Despite these limits, Halden and others believe that the information flowing through our sewers provides valuable insights into human health. “It’s like the microbiome,” he says. “The tools were there, but no one thought to look at all the microbes on our body. Now it’s a huge field.”

## Insights into Whole Communities

Work published in 2001^2^ by the EPA’s Daughton served as the foundation for sewage monitoring in estimating community-wide per capita exposure as well as estimating community health. In 2012 he proposed for the first time that endogenous biomarkers could be used to estimate actual levels of biological stress or disease in a defined population^35^—this is BioSCIM.

“Up until this work, the chemicals that were targeted in sewage monitoring were anthropogenic chemicals that were not suited to reflecting stress or health,” Daughton says. His work showed for the first time that it was possible to estimate health status as a combined function of all stressors, rather than just exposure to a select class of chemicals.

More recently, Daughton has focused on molecules called isoprostanes, which are markers of oxidative stress in the body.^36^ High levels of oxidative stress characterize a variety of acute and chronic conditions, including diabetes, heart disease, cancer, and obesity, and also reflect a wide variety of unhealthy practices (e.g., smoking) and lifestyles.^37^ Daughton hopes that measuring isoprostane levels (or levels of any of numerous other possible biomarkers) in the sewer systems can provide a snapshot of a community’s health. The feasibility of the analytical methodology required for monitoring sewage for isoprostanes was demonstrated in a 2015 report.^38^ However, he says, more work is necessary to prove the full feasibility of the approach.

Daughton also believes obesity trends could possibly be gauged by monitoring microbial signatures in wastewater—that’s because obese individuals tend to have different gut microbiome signatures than people of normal weight.^39^ Preliminary evidence reported in 2015 suggests this may in fact be the case.^40^ Researchers found that although microbial species from the human gut made up only about 15% of the bacterial DNA signatures in sewage, they captured 97% of the species found in human feces. Further analysis of their data revealed that they could predict with 81–89% accuracy whether the bacteria in their samples came from “lean” or “obese” cities (as determined by the percentage of obese residents).

“In the last 15 years, sewage is finally being looked at as a source of chemical information—what information can be mined, and how can we use it,” Daughton says.

And the field is just getting started. Researchers at the Massachusetts Institute of Technology recently undertook the development of “smart sewers” that may one day monitor the bacteria and viruses circulating in sewage to detect infectious disease outbreaks before they occur.^41^ In Europe, a research project called SEWPROF (short for A New Paradigm in Drug Use and Human Health Risk Assessment: Sewage Profiling at the Community Level)^42^ hopes to fund early-career scientists to improve wastewater analysis techniques and identify biomarkers for tracking human health. Another European initiative conducted through the European Cooperation in Science and Technology (COST)^43^ also seeks to improve community health by identifying sewage biomarkers.

Daughton says many additional applications will likely come to light in the future. “One example of another distinct application is the estimation of community population size in near real time—something that has never been possible before,” he says. “Real-time estimation of population size is critical to SCIM because it’s required to perform more accurate per capita back-calculations.”

For most people, encountering sewage remains a smelly and profoundly distasteful event, one that entails a call to the plumber and a pair of sturdy rubber gloves. But the smelly sewage so many of us write off could provide scientists with the ability to gauge human health at the population level for greatly reduced cost and in nearly real time. “This,” Daughton says, “would represent a paradigm shift in the tracking of public health trends and concerns.”

Halden puts it another way. “This is an information superhighway,” he says. “There is a huge flow of information that goes almost completely untapped because of our ignorance.”
